# All-microwave spectroscopy and polarization of individual nuclear spins in a solid

**DOI:** 10.1126/sciadv.adu0581

**Published:** 2025-03-07

**Authors:** Jaime Travesedo, James O'Sullivan, Louis Pallegoix, Zhiyuan W. Huang, Patrick Hogan, Philippe Goldner, Thierry Chaneliere, Sylvain Bertaina, Daniel Estève, Patrick Abgrall, Denis Vion, Emmanuel Flurin, Patrice Bertet

**Affiliations:** ^1^ Quantronics Group, Service de Physique de l’État Condensé (CNRS, UMR 3680), IRAMIS, CEA-Saclay, Université Paris-Saclay, 91191 Gif-sur-Yvette, France.; ^2^ University College London, LCN, QSD 17-19 Gordon Street, London WC1H 0AH, UK.; ^3^ Chimie ParisTech, PSL Université, CNRS, Institut de Recherche de Chimie Paris, 75005 Paris, France.; ^4^ Univ. Grenoble Alpes, CNRS, Grenoble INP, Institut Néel, 38000 Grenoble, France.; ^5^ Aix-Marseille Univ. University of Toulon, IM2NP, 13013 Marseille, France.

## Abstract

Pushing the sensitivity of nuclear magnetic resonance spectroscopy to the single spin level would have a major impact in chemistry and biology and is the goal of intense research efforts. We report magnetic resonance spectroscopy measurements of individual nuclear spins in a crystal coupled to a neighboring paramagnetic center, detected using microwave fluorescence at millikelvin temperatures. We observe real-time quantum jumps of the nuclear spin state, a proof of their individual nature. By driving the forbidden transitions of the coupled electron-nuclear spin system, we also achieve single-spin solid-effect dynamical nuclear polarization. Relying exclusively on microwave driving and microwave photon counting, the methods reported here are, in principle, applicable to a large number of electron-nuclear spin systems, in a wide variety of samples.

## Teaser

The method promises enhanced spectral resolution for probing the structure of matter in a large class of samples.

## INTRODUCTION

Measuring the magnetic resonance spectrum of individual nuclei would enable atomic-scale imaging of individual molecules with chemical sensitivity, but is made difficult by the small value of the nuclear magnetic moment (*1*–*3*). The sensitivity to directly detect as few as ∼100 nuclear spins has been achieved using mechanical (*1*, *2*, *4*) and atomic spin (*3*, *5*, *6*) sensors. So far, however, it has only been possible to measure individual nuclear spins through their hyperfine coupling to an individual paramagnetic electronic system, whose spin then needs to be detected. Certain electron spin systems can be detected optically using spin-dependent photoluminescence. This has enabled optical detection of individual ^13^C nuclear spins by nitrogen vacancy (NV) centers in diamond (*7*–*9*), of individual ^73^Ge nuclei by a GeV center in diamond (*10*), of individual ^13^C and ^29^Si by di-vacancy defects in silicon carbide (*11*), and of individual ^1^H (*12*) and ^29^Si (*13*) nuclei by rare earth ions in oxide crystals. In other systems, the electron spin can be converted into a charge, which can be detected using electrical currents. This has enabled electrical detection of individual ^31^P (*14*), ^29^Si (*15*), and ^123^Sb (*16*) nuclear spins by a phosphorus donor in silicon and of individual ^159^Tb nuclear spins in a TbPc_2_ single-molecule magnet (*17*). Electrical detection by a scanning tunneling microscope of the electron spin of individual atoms on a conducting surface enabled hyperfine spectroscopy of ^57^Fe, ^47^Ti, and ^49^Ti nuclear spins (*18*) and spectroscopy and polarization of ^63^Cu and ^65^Cu (*19*) nuclear spins.

Recently, individual electron spins were detected using microwave photon counting at 10 mK (*20*). This fluorescence detection method (*21*, *22*) relies solely on the magnetic coupling of the electron spin to a detection microwave resonator, whose role is to enhance the radiative spin relaxation rate $\Gamma_{R}$ via the Purcell effect (*23*, *24*). It is, therefore, in principle, applicable to a larger class of paramagnetic systems than optical or electrical detection. Here, we show that fluorescence detection of a single electron spin also enables readout, spectroscopy, and polarization of individual nuclear spins of the host crystal to which the electronic probe is strongly coupled.

## RESULTS

### Experimental setup and spin system

**Fig. 1.**
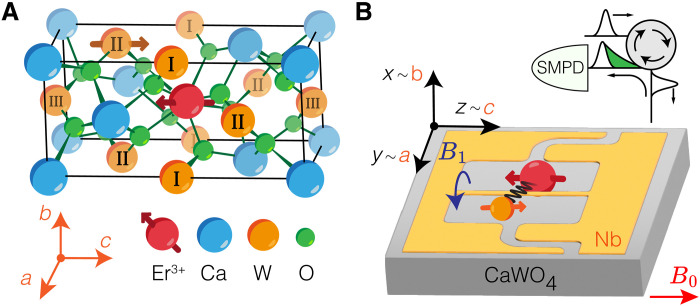
CaWO_4_ crystal structure and experiment schematic. (**A**) Schematic representation of the first unit cell of the CaWO_4_ crystal, which has tetragonal symmetry around the *c* axis. The crystalline axes (*a*, *b*, and *c*) are shown in orange. An Er^3+^ ion (red) substitutes a calcium ion (blue). Three types of W sites (labeled as I, II, and III) are distinguished on the basis of their location with respect to the Er^3+^. (**B**) Sample schematics. A resonator (yellow) is fabricated out of a niobium thin film on the surface of a CaWO_4_ slab (gray). The nanowire constriction generates an oscillating magnetic field $B_{1}$, which couples to an Er^3+^ electron spin in the vicinity (red). The latter may be coupled to one or several neighboring ^183^W nuclear spin (orange). The sample is connected to the microwave lines through an antenna. A circulator routes coherent excitation pulses (Gaussian curves) to the sample and the fluorescence signal (green exponential decay) to a single microwave photon detector (SMPD). The axes (*x*, *y*, and *z*) along the edges of the slab approximately match the crystalline axes (*a*, *b*, and *c*). A magnetic field $B_{0}$ is applied in the *yz* plane, approximately parallel to the wire.

For this demonstration, we use a crystal of CaWO_4_ (see Fig. 1A). The nuclear spin species with the largest concentration in the crystal is ^183^W, a 14.4% abundant isotope with a nuclear spin *I* = ^1^/_2_ and a low gyromagnetic ratio γ_*W*_/2π = 1.774 MHz T^−1^ (*25*). Individual ^183^W nuclear spins are detected by their hyperfine interaction with Er^3+^ ions randomly located throughout the crystal. The lowest-energy Kramers’ doublet of Er^3+^ ions forms an effective electron spin S = ^1^/_2_ with a gyromagnetic tensor γ (see section S1) when they enter CaWO_4_ in substitution to Ca^2+^ (*26*, *27*). A microwave resonator of frequency ω_0_/2π = 7.7492 GHz and linewidth κ/2π = 640 kHz is patterned directly on top of the CaWO_4_ sample (see Fig. 1B). The resonator is made out of a superconducting thin film of niobium and contains a narrow constriction around which the magnetic field *B*_1_ is strong, enabling fluorescence detection of the nearby Er^3+^ ions (see section S1). The resonator output is directed toward a single microwave photon detector (SMPD) based on a superconducting transmon qubit (*20*, *21*, *28*, *29*). For fluorescence detection, a microwave pulse of frequency ω_0_ is applied to the sample, and the number of counts *C* is then recorded during an integration time window. A magnetic field $B_{0}$ (amplitude $B_{0}$) is applied parallel to the sample surface, approximately along the crystalline *c* axis. When the frequency $\omega_{S}=|\gamma \cdot B_{0}|$ of an Er^3+^ ion is resonant with $\omega_{0}$, a peak in the ensemble-averaged $\left\langle C\right\rangle \left(B_{0}\right)$ is visible if the ion radiative relaxation rate $\Gamma_{R}$ is large enough (typically, larger than 10^3^ s^−1^). In our device, this condition is met for ions at a distance of ~150 nm or less from the resonator constriction (*20*) (see section S1).

The interaction between an Er^3+^ electron spin and a ^183^W nuclear spin can be described in the secular approximation by the Hamiltonian

$$H/\hbar =\omega_{S}S_{z}+\omega_{I}I_{z}+AS_{z}I_{z}+BS_{z}I_{x}$$ (1)

where $\omega_{I}=-\gamma_{W}B_{0}$ is the ^183^W Larmor frequency, A is the longitudinal hyperfine coupling arising from both dipolar magnetic and contact interactions, and B is the transversal hyperfine coupling whose origin is purely dipolar magnetic. Both A and B depend on the relative distance between the Er^3+^ and the ^183^W as well as their relative orientation with respect to the magnetic field $B_{0}$. Within the first unit cell and with $B_{0}$ applied parallel to the c axis, three sets of magnetically equivalent ^183^W atoms can be found, called types I, II, and III in the following (see Fig. 1A). All ^183^W nuclear spins beyond the first unit cell have lower couplings and are not resolved in this study.

**Fig. 2.**
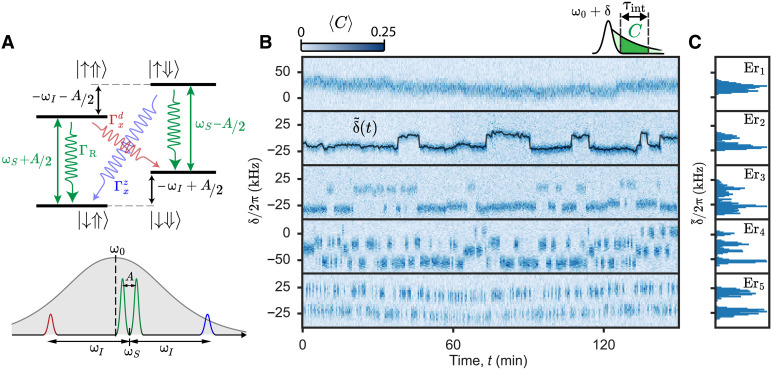
Single-^183^W-nuclear-spin quantum jumps. (**A**) Top: Energy level diagram of the ^183^W-Er^3+^–coupled system. Allowed electronic transitions are shown as straight green arrows. Relaxation rates are shown as wiggly arrows, green for allowed transitions (rate $\Gamma_{R}$), red for the double quantum (rate $\Gamma_{R}^{d}$), and blue for the zero quantum (rate $\Gamma_{R}^{z}$). Bottom: Schematic of the ^183^W-Er^3+^ transitions, superimposed with the resonator response shown as a gray Lorentzian of full width at half maximum κ. Allowed transitions (green peaks) are quasi-resonant with $\omega_{0}$, and forbidden transitions (red and blue) are detuned by $∼\omega_{I}$. (**B**) Top: Fluorescence detection spectroscopy pulse scheme. A Gaussian π-pulse of 80 μs of duration and frequency $\omega_{0}+\delta$ is applied to the sample. Ensemble-averaged number of counts ⟨C⟩ is subsequently measured during an integration time window, τ_int_. The sequence is then repeated without any delay, with the excitation pulse frequency updated. Bottom: High-resolution spectra of five Er^3+^ ion spins, measured consecutively for 150 min. Each spectrum is recorded by averaging 200 sweeps of δ, yielding the ensemble-averaged number of counts ⟨*C*⟩(δ). The integration time τ_int_ is 1.6 ms for Er_1_, 2.0 ms for Er_2,3_, and 3.6 ms for Er_4,5_. The increase in counts corresponds to the detected fluorescence after exciting an electron paramagnetic resonance (EPR)–allowed transition. The resonance frequency evolves in time with a slow drift and sudden telegraphic jumps. Each spectrum is fitted as a Lorentzian and the center $\tilde{\delta }$ is shown as a function of time for Er_2_ as a black continuous line. (**C**) Histograms of $\tilde{\delta }$ for the five ions.

In the high-field limit $|\omega_{I}|\gg A\;\text{and}\;B$, the eigenstates of the two-spin system are close to the uncoupled spin states ∣↑⇓⟩, ∣↓⇓⟩,∣↑⇑⟩, and ∣↓⇑⟩, in ascending order of energy, as shown schematically in Fig. 2A. Because $\left\langle ↓⇑|S_{x}|↑⇑\right\rangle \approx \left\langle ↓⇓|S_{x}|↑⇓\right\rangle \approx$ ^1^/_2_, the two nuclear-spin preserving transitions at $∼\omega_{S}\mp A/2$ are electron paramagnetic resonance (EPR) allowed. They can be driven by a resonant microwave pulse; moreover, cavity-enhanced radiative relaxation at rate Γ*_R_* can take place on either transition, giving rise to the fluorescence detection signal. On the other hand, $|\langle ↓⇓|S_{x}|↑⇑\rangle |\approx |\langle ↓⇑|S_{x}|↑⇓\rangle |\approx |B/\left(4\omega_{I}\right)|$, implying that the nuclear-spin flipping transition at $\omega_{S}+\omega_{I}$ (respectively, $\omega_{S}+\omega_{I}$) is not completely forbidden when B is nonzero (*30*). This double-quantum (respectively, zero-quantum) forbidden transition can, thus, be microwave driven. Moreover, radiative electron relaxation with nuclear spin flipping (called cross-relaxation in the following) also becomes weakly authorized, at a rate $\Gamma_{x}^{d,z}=\Gamma_{R}\frac{B^{2}}{4\omega_{I}^{2}}\frac{1}{1+4{\left[\omega_{I}\pm \left(\omega_{S}-\omega_{0}\right)\right]}^{2}/\kappa^{2}}$. The last factor is due to the off-resonant Purcell effect from the superconducting resonator. We note that the two cross-relaxation rates are equal only if the spin and cavity are on resonance ($\omega_{S}=\omega_{0}$). We also introduce the cross-relaxation probability $\eta^{d,z}\equiv \Gamma_{x}^{d,z}/\left(\Gamma_{R}+\Gamma_{x}^{d,z}\right)\approx \Gamma_{x}^{d,z}/\Gamma_{R}$

### Nuclear spin quantum jumps

We isolate individual Er^3+^ ion spins with $\Gamma_{R}$ ∼ 10^3^ s^−1^ by scanning the field amplitude $B_{0}$ in a region around 446 mT (close to the Er^3+^ *I* = 0 main resonance line) and identifying single peaks as explained in (see section S9) (*20*). We perform high-resolution spectroscopy by exciting the spin with an 80-μs–long Gaussian-shaped microwave π-pulse, with frequency ω followed by microwave fluorescence detection, sweeping $\delta =\omega -\omega_{0}$ over 2π × 100 kHz. Each sweep is averaged 200 times to obtain the ensemble-averaged number of counts ⟨C⟩(ω). Figure 2B shows such spectra measured repeatedly for 150 min, for five representative ions labeled as Er_1_ to Er_5_. Ion Er_1_ displays a single line, whose center frequency fluctuates on a timescale of several minutes and on a frequency scale of ∼20 kHz. This spectral diffusion is attributed to a combination of $B_{0}$ drift, charge noise, and rearrangements of the weakly coupled nuclear spin bath and was observed for all measured ions although with a different intensity (see discussion below). Because the drift is slow, it can be compensated by various frequency-tracking strategies (see section S3), which are used in all the measurements reported later in this work. In contrast, the electron spin frequencies of ions Er_2_, Er_3_, and Er_5_ display a clear telegraphic noise between two frequencies, four in the case of Er_4_. As demonstrated in the following, these jumps occur when one nearby ^183^W nuclear spin changes state. Quantum jumps are a hallmark of the measurement of individual quantum systems and have been observed for trapped ions (*31*–*33*), photons in a high-*Q* microwave cavity (*34*, *35*), superconducting circuits (*36*), and various nuclear spin systems (*7*, *14*, *37*); here, we report their observation on individual ^183^W nuclear spins by microwave fluorescence detection.

The transition frequency of each spectrum $\tilde{\delta }\left(t\right)$ is obtained from a Lorentzian fit, shown as a histogram in Fig. 2C for the five ions. Each histogram presents a different number of resolved distributions. For Er_2_, Er_3_, and Er_5_, the two resolved distributions indicate the presence of a strongly coupled neighboring nuclear spin. The size of the jumps is a direct measure of the hyperfine coupling constant |A| (see section S4). In contrast, Er_4_ shows four resolved distributions that correspond to two strongly coupled nuclear spins. The pair will be referred as Er_4_^(1)^ and Er_4_^(2)^ in the following. We note that the standard deviation of the individual distributions is notably different for each ion, indicating that part of the drift originates from their local environment, likely from unresolved nuclear spin jumps.

The direct relaxation rate of ^183^W nuclear spins proximal to Er^3+^ impurities in CaWO_4_ is exceedingly slow at 10 mK, with an upper bound of ∼10^−6^ s^−1^ (*38*). We therefore attribute all observed quantum jumps to cross-relaxation events occurring via the Er^3+^ spin excited state, enabling to determine cross-relaxation probabilities $\eta^{d,z}$ from each ion time trace (see table S2). A substantial difference between $\eta^{d}$ and $\eta^{z}$ is observed for Er_2_,_3_,_4_, as also evidenced by the different peak weights in the histograms of $\tilde{\delta }$. The spin-resonator residual detuning (less than 30 kHz) is too low to explain this asymmetry in the radiative cross-relaxation model. This may indicate the existence of an extra, non-radiative, asymmetric cross-relaxation channel, possibly arising from the coupling to another detuned Er^3+^ ion (*39*). From the lowest value of $\eta^{d,z}$, we use the expressions above and extract B for each nuclear spin (shown, together with A, in table S1). Based on calculated values of A and B assuming a purely dipolar interaction, we tentatively assign the nuclear spins Er_2_ and Er_4_^(2)^ to type III sites, the nuclear spin of Er_5_ to a type I site, and the nuclear spin of Er_3_ and Er_4_^(1)^ to either a type I or a type II site (see section S8).

### Nuclear spin state readout

**Fig. 3.**
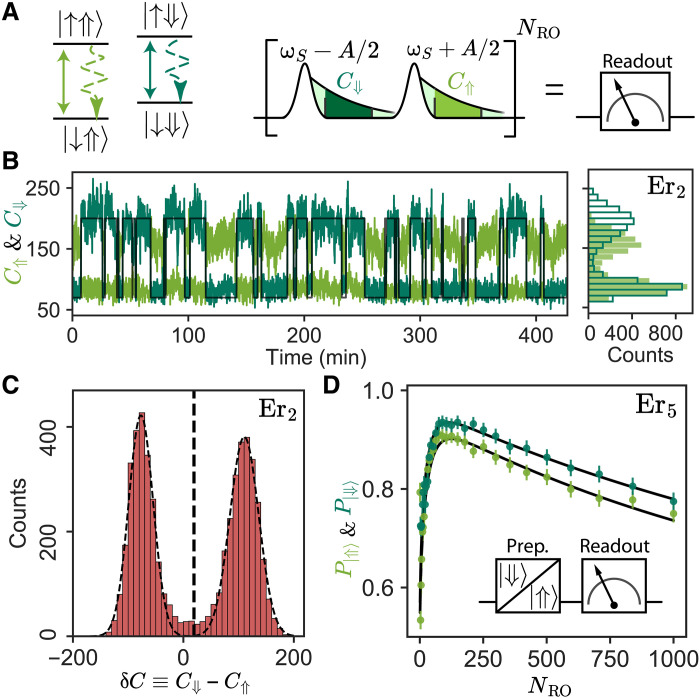
Single-shot ^183^W nuclear spin readout. (**A**) Left: Energy level diagram of an ^183^W-Er^3+^–coupled system. EPR-allowed transitions are represented as solid bright and dark green lines, and the respective radiative decays in dashed lines of the same color. Right: Single-shot nuclear readout pulse sequence. Resonant pulses are applied on each EPR-allowed transition, and the number of counts is recorded independently for the two frequencies. The sequence is repeated *N*_RO_ times, yielding the number of counts after each sequence, $C_{⇓}$ and $C_{⇑}$. (**B**) Left: Integrated counts $C_{⇓}$ (respectively, $C_{⇑}$) as a function of time as a dark green (respectively, light green) solid line. The data were taken for Er_2_ with integration time $\tau_{\text{int}}$ = 1.6 ms and $N_{\text{RO}}$ = 10^3^. Quantum jumps appear as abrupt changes in the number of counts with telegraphic trajectory. The two traces are anticorrelated and the state of the nuclear spin can be mapped to ∣⇓⟩ when $C_{⇓}>C_{⇑}$ represented by a black continuous line. Right: Histograms of $C_{⇓}\;\text{and}\;C_{⇑}$. The lower number of counts in state ⇑ is attributed to imperfect SMPD frequency tuning, resulting in different fluorescence intensities. (**C**) Histogram of $\delta C\equiv C_{⇓}-C_{⇑}.$ A bimodal Gaussian distribution fit is plotted as black dashed line. Vertical black dashed line shows the threshold for single-shot readout. (**D**) Probability of measuring state ∣⇓⟩ and ∣⇑⟩ (dark and light green respectively) as a function of $N_{\text{RO}}$, after preparation in the respective state. Experimental data are shown as green dots, and the fit is shown as a solid black line (see section S5). The data were obtained for Er_5_ with integration time $\tau_{\text{int}}$= 2 ms. Inset: Pulse sequence. The nuclear spin is initialized into state ⇓ (respectively, ⇑) followed by state readout sweeping $N_{\text{RO}}$.

We leverage the ability to spectrally resolve the different allowed transition frequencies of the electronic spin to perform a projective, quantum–non-demolition measurement of the nuclear spin state. We apply sequences of frequency-resolved excitation pulses (Gaussian-shaped, 80 μs long) at $\omega_{S}-A/2$ and $\omega_{S}+A/2$, spaced by an integration time $\tau_{\text{int}}$ during which the fluorescence counts are measured; repeating this $N_{\text{RO}}$ times yields the number of counts after each sequence $C_{⇑}$ and $C_{⇓}$ (see Fig. 3A). Time traces over 7 hours for these two quantities taken with ion Er_2_ are shown in Fig. 3B, using $N_{\text{RO}}$ = 10^3^ readout pulses. The two traces are clearly anticorrelated. The lower signal level in $C_{⇑}\left(t\right)$ compared to $C_{⇓}\left(t\right)$ is attributed to imperfect tuning of the SMPD center frequency. The photon-counting histograms for each time trace show two well-separated distributions. From the histogram of $\delta C\equiv C_{⇓}-C_{⇑},$ which shows two separated and symmetric peaks, we define a threshold enabling single-shot readout of the nuclear spin state. Data shown in Fig. 3 (C and D) are for ion Er_2_, for which |A∣/2π = 21 kHz.

We prepare the nuclear spin in state ∣⇓⟩ (respectively, ∣⇑⟩) (see below) and measure $P_{|⇓\rangle }$ (respectively, $P_{|⇑\rangle }$) as a function of the number of readout pulses, $N_{\text{RO}}$, for ion Er_5,_ for which |A∣/2π = 40 kHz. At first, the probability of a correct readout increases with $N_{\text{RO}}$, due to the increase in signal-to-noise ratio. After reaching a maximum value, $P_{|⇓\rangle }$ then slowly decreases with $N_{\text{RO}}$, due to the finite cross-relaxation probability $\eta^{d,z}$ at each readout step. The analytical model used to describe the two mechanisms (see section S5) reproduces quantitatively the observations and yields an estimated value for the transversal hyperfine coupling of B/2π ∼ 70 ± 7 kHz, close to the value extracted from the time traces. This indicates that cross-relaxation is the limiting factor for nuclear spin readout fidelity. It could be suppressed further in future experiments by increasing the resonator quality factor, hence the cavity filtering.

### Single nuclear spin spectroscopy

**Fig. 4.**
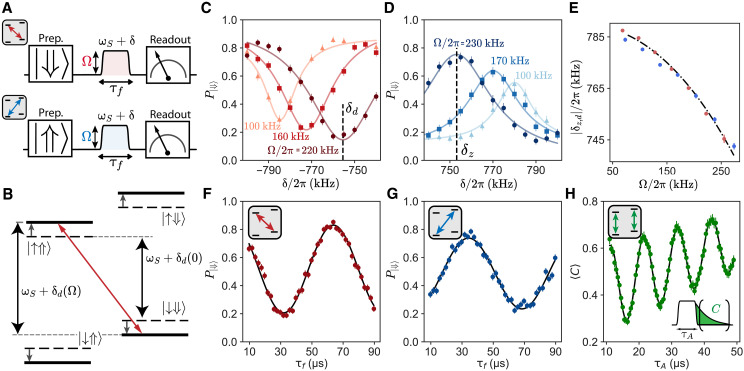
Single-spin ELDOR-detected NMR spectroscopy. (**A**) Pulse sequence. To measure the double-quantum (respectively, zero-quantum) transition, the nuclear spin is first prepared in the ⇓ (respectively, ⇑) state. A microwave pulse with a flattop-Gaussian envelope (maximum amplitude Ω) and a frequency $\omega_{S}+\delta$ is applied, followed by nuclear-spin readout. (**B**) ac-Zeeman shift diagram. The spin levels are frequency shifted by the microwave drive, from their Ω = 0 value (black dashed lines) to the value under drive (solid black line). (**C** and **D**) Measured (dots) nuclear spin ⇓ probability as a function of δ for three values of Ω with pulse duration $\tau_{f}$ equal to 60, 32, and 22 μs in increasing order of Ω. Panel (C) [respectively, (D)] shows the double-quantum (respectively, zero-quantum) transition. Solid lines are Lorentzian fits, yielding the measured transition frequency $\delta_{d}$ (respectively, $\delta_{z}$) (**E**) Measured double-quantum (respectively, zero-quantum) frequency |$\delta_{d}$| (respectively, |$\delta_{z}$|) as a function of Ω. Dash-dotted line is a fit, using the ac-Zeeman shift model described in section S6. (**F** and **G**) Measured nuclear spin ⇓ probability as a function of $\tau_{f}$ for the double-quantum [(F), red dots] and zero-quantum [(G), blue dots] sequences, with δ set at resonance, for Ω/2π = 220 kHz. Black lines are cosine fits, yielding a forbidden Rabi frequency $\Omega_{z,d}/2\pi$ ∼ 15 kHz for both. Insets: Diagram representing which transition is being driven. (**H**) Allowed transition Rabi oscillation. A pulse at $\omega_{S}$ of duration $\tau_{A}$ is applied followed by fluorescence detection. The input pulse amplitude is 6.2 times lower than in (F) and (G). Green dots are ensemble-averaged number of counts ⟨C⟩ as a function of $\tau_{A}$. Black line is a cosine fit with linearly increasing offset, yielding Ω/2π = 97 kHz. Inset: Top: Transition diagram. Bottom: Pulse sequence. Data obtained from Er_5_.

Nuclear spin spectroscopy is achieved by driving the forbidden transitions, in a single-spin version of the electron double resonance (ELDOR)–detected nuclear magnetic resonance (NMR) experiment (*40*). Following preparation in state ∣⇓⟩ (respectively, ∣⇑⟩) (see below), a flattop-shaped pulse is applied at a frequency $\omega_{S}+\delta .$ The maximum amplitude of the pulse, Ω, is quantified by the Rabi frequency of the allowed transitions (see Fig. 4, A and B). A reduction (respectively, increase) in the nuclear spin state probability $P_{|⇓\rangle }$ is observed when $\omega_{S}+\delta$ matches the forbidden double-quantum (respectively, zero-quantum) transition. Data are shown in Fig. 4C (respectively, D) for three values of Ω, showing a frequency shift and power broadening.

The fitted frequencies for the double- and zero-quantum transitions, $\delta_{d}$ and $\delta_{z}$, respectively, are shown in Fig. 4E as a function of Ω. $|\delta_{d,z}|$ are found to depend quadratically on Ω. This is due to off-resonant driving of the allowed transitions, which leads to ac-Zeeman shifts of the energy levels (see Fig. 4B). In the high-field limit, $|\delta_{d,z}|\approx -\omega_{I}-\frac{\Omega^{2}}{2|\delta_{d,z}|}$. The combined values of $|\delta_{d}|$ and $|\delta_{z}|$ are fitted to the complete analytical expression (see section S6), yielding the nuclear spin Larmor frequency, $\omega_{I}/2\pi$ = −788.1 ± 4 kHz. The corresponding gyromagnetic factor of 1.767 ± 1 MHz T^−1^ is close to the value expected for ^183^W in CaWO_4_, $\gamma_{W}/2\pi$ = 1.774 MHz T^−1^ (*25*), thus confirming the nature of the nuclear spin.

Driving the forbidden transitions provides an alternative method to determine ***B***. The zero-quantum (respectively, double-quantum) Rabi frequency $\Omega_{z}$ (respectively, $\Omega_{d}$) is related to the allowed transition Rabi frequency as $\Omega_{z,d}\approx \Omega \frac{B}{2\omega_{I}}$. Double- and zero-quantum Rabi oscillations are shown in Fig. 4 (F and G) and allowed Rabi oscillations in Fig. 4H. The value obtained by this method, ∣B∣/2π = 103 ± 7 kHz, is ∼30 kHz larger than the value previously determined, possibly pointing to an underestimation of the uncertainties in the cross-relaxation method (see section S7).

### Single nuclear spin polarization

**Fig. 5.**
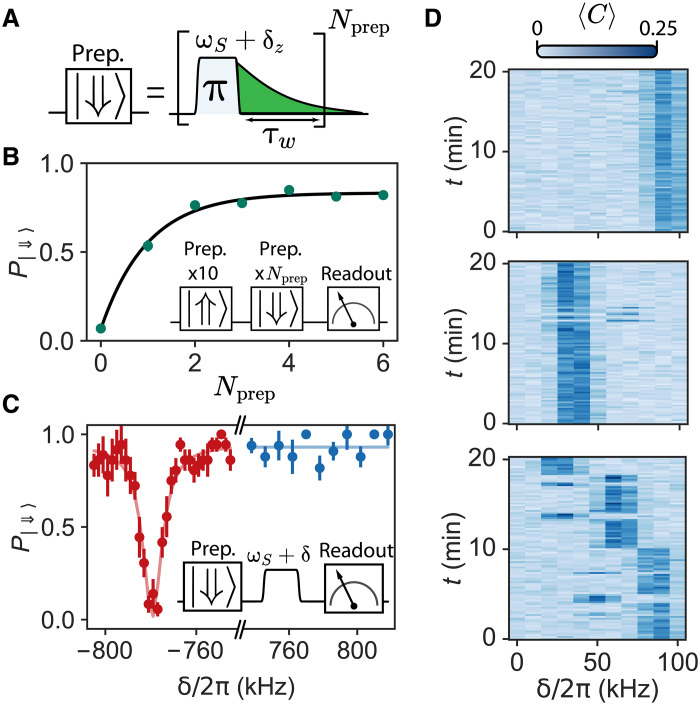
Single-spin dynamical nuclear polarization via solid effect. (**A**) Pulse sequence for preparing the nuclear spin in ⇓ (respectively, ⇑) through solid-effect dynamic nuclear polarization (DNP). One π-pulse at $\omega_{S}+\delta_{z}$ (respectively, $\omega_{S}+\delta_{d}$), followed by a relaxation time $\tau_{w}$, prepares the nuclear spin state to the desired state. The sequence is repeated $N_{\text{prep}}$ times for higher efficiency. (**B**) Measured (dots) nuclear spin ⇓ probability as a function of the number of preparation pulses $N_{\text{prep}}$ into this state. Data obtained for Er_5_. Solid line shows an exponential fit. Inset: Pulse sequence diagram. The nuclear spin is initialized into the ⇑ state. Then, the preparation sequence for different $N_{\text{prep}}$ and $\tau_{w}$ = 3 ms is applied. Last, the state of the nuclear spin is measured. (**C**) Probability of measuring the nuclear spin in state ⇓ in an ELDOR-detected NMR experiment for the zero- and double-quantum transitions after preparation in the ⇓ state. Inset: ELDOR-detected NMR sequence. $N_{\text{prep}}$ = 10 pulses and $\tau_{w}$ = 5 ms are used for preparation. Data obtained for Er_4_. (**D**) Ensemble-averaged counts ⟨C⟩ measured in a fluorescence detection spectroscopy experiment as a function of frequency during 20 min. Data obtained for Er_4_, which has two neighboring nuclear spins. From top to bottom, the nuclear spins are prepared to ∣⇑⇑⟩ and ∣⇓⇓⟩ and not prepared. When preparing the state, the electron spin frequency $\tilde{\delta }$ remains constant (except three spectra where preparation was unsuccessful). In the absence of state preparation, the frequency $\tilde{\delta }$ changes after each quantum jump.

The nuclear spin state can be initialized via solid-effect dynamic nuclear polarization (DNP) (*41*–*43*). To initialize the nuclear spin in ∣⇑⟩ (see Fig. 5A), we apply a pulse on the zero-quantum transition, wait for a time **τ_w_** (taken to be close to $\Gamma_{R}$^−1^), and repeat $N_{\text{prep}}$ times. $P_{|⇓\rangle }$ increases rapidly, reaching ≈ 0.8 for two pulses (Fig. 5B). This demonstrates single-atom solid-effect nuclear spin polarization. The maximum value is limited by nuclear-spin readout errors and out-of-equilibrium excitations of the electron spin and can likely be improved in future experiments.

State preparation is also confirmed spectroscopically (Fig. 5C). After polarizing the state of the nuclear spin to ∣⇓⟩, a pulse is applied at a frequency $\omega_{S}+\delta$, with the amplitude corresponding to a π-pulse for the forbidden transition at resonance, followed by nuclear spin state readout. The probability $P_{|⇓\rangle }$ shows a clear dip at the frequency corresponding to the double-quantum resonance, but no dip on the zero-quantum transition, which is further evidence for nuclear spin polarization.

The solid-effect DNP is also effective when multiple spins are present, as seen in Fig. 5D where it is applied to Er_4_. The polarization protocol into ∣⇓⇓⟩ involves sending pulses at each of the zero-quantum transition frequencies. A 20-min time trace of the allowed transition spectrum is shown in Fig. 5D in the absence of state preparation (bottom) and with polarization into ∣⇓⇓⟩ (middle) and into ∣⇑⇑⟩ (top). The polarization sequence is applied at the beginning of every spectrum. Whereas the trace with no polarization shows nuclear spin quantum jumps as reported in Fig. 2B, the two other traces show dominantly the one allowed transition corresponding to the polarized state.

## DISCUSSION

A logical next step will be to extend these methods to nuclear spins more weakly coupled to the electron spin, using methods demonstrated with NV centers in diamond (*6*, *8*, *9*). This would open the way to ^183^W-nuclear-spin–based quantum registers operated and readout via the Er^3+^ spin, generalizing recent ^13^C quantum register demonstrations operated by an NV center in diamond (*44*, *45*). Perhaps the most interesting aspect of the methods reported here is their applicability to a large range of nuclear spin systems in a large variety of samples, crystalline or not. Individual paramagnetic centers can be detected by microwave fluorescence provided their Purcell relaxation rate $\Gamma_{R}$ can be increased above ∼10^3^ s^−1^ and above their non-radiative relaxation rate at 10 mK. These conditions can likely be met in a variety of paramagnetic systems, ranging from organic radicals to transition-metal ions in metallo-enzymes, which could be deposited on top of the resonator constriction in the form of small molecular crystals or even frozen solutions. Applying the techniques demonstrated here may then yield their hyperfine couplings to individual ligand nuclear spins with a higher spectral resolution than reached with ensemble measurements.

## MATERIALS AND METHODS

### Material characteristics

The CaWO_4_ sample was cut out of a boule grown at Institut de Recherche de Chimie Paris in a rectangular slab shape, same as the one used in (*20*). Its dimensions are 7 mm by 4 mm by 0.5 mm, with its surface approximately along the *ac* plane of the crystal and the *c* axis along the short edge. The gyromagnetic tensor of Er^3+^ is diagonal in the (*a*, *b*, *c*) coordinate system, $\gamma_{a}=\gamma_{b}=\gamma_{⊥}=-2\pi \cdot 117.3$ GHz/T and $\gamma_{c}=\gamma_{∥}=-2\pi \cdot 17.45$ GHz/T (*46*). Three resonators with a frequency separation of 150 MHz are fabricated on the sample.

### Resonator characteristics

Without any applied field, the resonator’s frequency is 7.774 GHz, $Q_{\text{ext}}$ = 1.6 · 10^4^ and $Q_{\text{int}}$ = 4.0 · 10^4^. The total quality factor is *Q* = 1.14 · 10^4^.

With $B_{0}$ = 446 mT approximately parallel to the crystalline *c* axis, the resonator’s frequency is 7.748 GHz, $Q_{\text{ext}}$ = 2.0 · 10^4^ and $Q_{\text{int}}$ = 2.8 · 10^4^. The total quality factor under field is *Q* = 1.16 · 10^4^.

### Resonator fabrication

The superconducting resonator was fabricated on top of the crystal. The process starts with the sputtering of a 50-nm thin film of niobium on top of the CaWO_4_ sample. A 30-nm aluminum mask, in the resonator shape, is deposited on top of the niobium with a liftoff process. The unmasked niobium is removed through dry etching using a 1:2 mix of CF4 and Ar gas. Last, the Al mask is removed in a MF-319 solution.
