# Supplementary material for: All-microwave spectroscopy and polarization of individual nuclear spins in a solid
Source: Sci Adv. 2025 Mar 7;11(10):eadu0581. doi: 10.1126/sciadv.adu0581 (PMC11887841; doi:10.1126/sciadv.adu0581)
Supplement: Supplementary file 1 — Supplementary Text Figs. S1 to S8 Tables S1 to S3 [file sciadv.adu0581_sm.pdf]

Science Advances
AAAS

Supplementary Materials for

All-microwave spectroscopy and polarization of individual nuclear spins in a solid

Jaime Travesedo *et al.*

Corresponding author: Patrice Bertet, patrice.bertet@cea.fr

Sci. Adv. **11**, eadu0581 (2025)
DOI: 10.1126/sciadv.adu0581

This PDF file includes:

Supplementary Text
Figs. S1 to S8
Tables S1 to S3

Supplementary Materials

1. Sample and experimental setup

The frequencies of the fabricated resonator were measured and reworked by etching part of the capacitance to lie in the SMPD operating range (7.70 - 7.76 GHz). During the rework process of the sample used for this experiment, the crystal broke and two out of the three resonators were destroyed. Figure S1A shows a stitched, false color micrograph of the remaining resonator.

The sample is hosted in a 3D copper cavity with a single port which couples capacitively to the resonator through a microwave antenna The full wiring diagram is shown in Fig. S2. The resonator uses a the same "bowtie" design as described in (*20*), albeit with a reduced 0.3 x 50 µm wire. The reduced dimensions of the wire result in an increased coupling between the Er^{3+} ions and the resonator, represented as a 2D map in S1B. The sample holder is thermalized at 10 mK in a dilution cryostat, and placed at the center of a vector 1/1/1 T magnet. The magnetic field B_0 must be applied in the resonator *(a, c)* crystalline plane, since out-of-plane components will rapidly reduce the quality factor and destroy the superconducting properties of the resonator. We align the magnetic field in the resonator plane, by using the resonator response as described in (*20*). However, the resonator plane is not exactly aligned with the *(a, c)* plane. We define β the angle between the resonator plane and the *c*-axis, and θ the angle between the *c*-axis projection and the applied magnetic field (see Fig. S1C)

2. SMPD characteristics

All measurements were performed with the use of a Single Microwave Photon Detector (SMPD). Succinctly, the arrival of a microwave photon at the device input is mapped onto the excited state of a superconducting transmon qubit by a parametric process activated by a pump tone. The qubit is dispersively read-out cyclically and counts are recorded when found in the excited state. More details about the design and previous versions of the device can be found in (*21,29*). The counter used in the experiment was operated with a bandwidth of ~ 300 kHz for which it presents a maximum efficiency of 0.79(1) and a dark count rate of 40(5) counts per second. The average duration of a complete measurement cycle is ~ 17 µs, with a certain variation due to the active reset performed on the qubit, which can extend the sequence. The down-time of the detector, during which the qubit is read-out, is ~ 2 µs per cycle.

The operational dark-counts, Γ_{DC}, and the spin-efficiency ϵ, probability of measuring a photon coming from the spin sample, were measured through the fluorescence decay of a single spin (see Fig. S3). The average number of photons detected from a spin signal is measured by integrating the background-removed fluorescence curve. Since the number of spins being excited is one, the average number of expected photons is equivalent to the spin-efficiency. Furthermore, the signal will present a flat background count-rate corresponding to the dark counts of the detector, coming from spurious excitations in the qubit and heat dissipation on the microwave lines. During the measurements, the spin-efficiency ranged from 0.2 to 0.4 and the dark count rate spanned from 40 to 150 counts per second, depending on the quality of the calibration of the detector. The spin

efficiency does not reach the maximum efficiency of the SMPD due to the internal losses of the resonator.

3. Tracking algorithms

As is visible in Fig.2 of the main text, the allowed EPR frequencies of individual erbium ions show fluctuations over time, due to a combination of B_0 drifts, nuclear spin environment dynamics, and charge noise. The measured drift is comparable to the linewidth of the EPR-resonance of the Er^{3+} ions. However, the drift is slower than cross-relaxation events and can be compensated for. Two different tracking algorithms were implemented, one based on direct spectroscopy and the other a continuous Proportial-Integral (PI) loop.

The first algorithm performs high-resolution spectroscopy with an 80 μs π-pulse in a 50 kHz range centered around the expected transition frequency. If there are multiple possible transitions due to nearby nuclear spins, the nuclear spins are initialized into a predetermined state before the spectroscopy. The ensemble averaged number of counts $C(\delta)$ is then fitted to a Lorentzian model, as shown in Fig. S4A. From the center of the fit, the drift is calculated. The measurement takes 1 minute to complete. Due to its long duration, it can only be run a limited number of times, which results in a poor tracking.

The second method uses a PI-loop to track the value of one of the electron spin transitions. A diagram explaining the control sequence is shown in S4B. After performing a measurement, noise from the environment induce random drifts. This drift can be characterized through a Ramsey experiment. If the drive frequency is detuned $\Delta\omega$ from the transition frequency, the state of the system after a Ramsey sequence is $\langle S_z \rangle = e^{-t/T_2^x} \cdot \sin(\Delta\omega \cdot \tau)$. The number of fluorescent counts C gives a proxy for $\langle S_z \rangle$, making this sequence a good sensor for $\Delta\omega$. However, the presence of fluctuating dark counts in the measurement makes defining a setpoint for the PI controller a hard task. As such, we effectively remove this problem by performing the negative phase Ramsey sequence and subtracting the measured number of counts $\bar{C}$ from the original value. The two measurements are interleaved and performed $N_{track} = 10$ times. The quantity $C - \bar{C}$ is a good sensor since it has a stable setpoint at 0 and depends linearly with the detuning, as long as $\tau \ll 2\pi/\Delta\omega$. To increase the convergence speed, we allow the system to have a short memory time by filtering the values of the sensor output for every iteration i of the PI loop to generate the filtered sensor Y_i

$$Y_i = \sum_{j=0}^{i} \left(C_j - \bar{\bar{C}}_j\right) \cdot e^{\frac{-(j-i)}{f}} \approx \left(1 - \frac{1}{f}\right) \cdot Y_{i-1} + \left(C_i - \bar{\bar{C}}_i\right) \tag{S1}$$

Where f = 2000 is the memory of the system. Finally, the PI controller calculates the frequency correction to be applied on the pulses through a proportional and an integral channel

$$\delta\omega_i = P \cdot Y_i + I \cdot \sum_{j=0}^{i} Y_j. \tag{S2}$$

The values for P and I were set by manually optimizing the convergence time after artificially detuning the pulse frequency. As for the other sequence, we initialized any resolved nuclear spins into a predefined state before the tracking is performed.

4. Time trace analysis

The traces were obtained by performing high-resolution spectroscopy with an 80 µs long Gaussian pulse and sweeping the frequency over 100 kHz, then the ensemble average number of counts is recorded. The duration of the detection window after the pulse is set to approximately the T_1 of the spin. After the pulse is sent, we wait between 60 and 120 μs to avoid spurious counts due to the heating of the microwave lines. Each curve from the trace is calculated by averaging 200 frequency sweeps. A trace for Er_5 is presented in Fig S5A. Each curve is fitted with a multi-Lorentzian model and the center of the most prominent resonance is defined as $\tilde{\delta}$ (Fig. S5B). To determine the state of the nuclear spin state for each moment in time, all the values of $\tilde{\delta}$ are sorted in increasing order and thresholds are calculated based on a derivative bound (see Fig. S5C). The state of the nuclear spin is then classified based on the frequency of the resonance (see Fig S5D). We also define $\Delta\tilde{\delta}$, as the difference between consecutive values of $\tilde{\delta}$, $\Delta\tilde{\delta}(t) \equiv \tilde{\delta}(t) - \tilde{\delta}(t - \Delta t)$.

Histograms of $\Delta\tilde{\delta}$ for $Er_{1\text{-}5}$ are shown in Fig. S5E, which consist of several peaks. The peak centered at 0 arises when no resolved jump occurs in-between two consecutive traces. A Gaussian fit yields a Full-Width-Half-Maximum varying from 4.5 to 10 kHz, indicating that the short-term noise (at the minute scale) also varies notably from ion to ion. The extra peaks observed for $Er_{2\text{-}5}$ arise from resolved nuclear spin quantum jumps. Gaussian fits to these peaks yield the mean frequency jump size which we identify to the longitudinal hyperfine coupling A, and the jump standard deviation which we find similar to the central peak, as expected.

Using the state assignment, the cross-relaxation probabilities $\eta^{d,z}$, is measured. $\eta^{d,z}$ is defined as

$$\eta^{d,z} \approx \frac{\Gamma_x^{d,z}}{\Gamma_R} \approx \frac{N_x^{d,z}}{N_{\text{exc}}^{\Downarrow,\Uparrow}}, \tag{S3}$$

where $N_x^{d,z}$ is the number relaxation events through the zero- and double-quantum transition and can be directly counted from the now state classified traces. $N_{\text{exc}}^{\Downarrow,\Uparrow}$ is the number of excitations of the electron spin when the nuclear spin is in the $\Downarrow$ (resp. $\Uparrow$) state. This quantity is directly proportional to the number of curves classified in the respective nuclear spin state, while taking into consideration the relative amplitudes of the multi-Lorentzian fit. This value is then multiplied by the number of averages of each spectrum. However, due to the non-negligible spectral width of the Gaussian pulse, each sweep does not excite the electron spin exactly one time. Based on the shape and length of the pulse as well as the frequency step of each measurement, an approximate number of excitation pulses is calculated from the Fourier transform of the pulse. Table I presents the cross-relaxation probabilities for the coupled nuclear spins of $Er_{2\text{-}5}$.

In the case of Er_2 and Er_4 we measure a notable difference between η^d and η^z, the former being almost double than the latter. This is consistent with the behaviour that the nuclear spins display in the time trace, spending a considerably longer amount of time in the $\Downarrow$ state. To understand the

origin of this difference, more in-detail measurements of the system are required. A possible explanation is the existence of an extra, non-radiative relaxation channel.

5. Read-Out probability fit

The probability of correctly assigning the state of the nuclear spin after N_{RO} measurements is obtained from a combination of two effects: the increase of the signal-to-noise ratio (SNR) as a function of the number of measurements and the evolution of the nuclear spin states populations due to cross-transition events.

Let us consider the measurement of a single nuclear spin that is completely polarized in $|\Downarrow\rangle$. As detailed in the main text, $C_{\Uparrow}$ and $C_{\Downarrow}$ are the total number of counts measured after applying a π −pulse and an SMPD measurement N_{RO}times at the two EPR-allowed frequencies of the electron spin. Since the nuclear spin is completely polarized, the only contributions to $C_{\Uparrow}$ will be the dark counts of the detector, which results in a Poissonian distribution with rate $\lambda = N_{RO} \cdot \Gamma_{DC} \cdot t_D$ where Γ_{DC} is the dark count rate of the detector and t_D is the duration of the detection window. On the other hand, $C_{\Downarrow}$will be the result of two processes, the aforementioned Poissonian events due to dark counts and the real detection events, which follow a binomial distribution with $n = N_{RO}$ and probability $p = \epsilon$, where ϵ is the probability of detection. The direct addition of the two distributions is not straightforward, however, both the Poisson and binomial distributions can be approximated to a Gaussian distribution when $\lambda >> 1$ and $n >> 1$ respectively. Under this approximation the addition of the two Gaussian distributions is trivially performed by adding the means and variances. The distributions for $C_{\Uparrow}$ and $C_{\Downarrow}$ are then

$$\begin{gathered} C_{\Uparrow} \sim \mathcal{N}(\mu_{DC}, \sigma_{DC}^2) \\ \mu_{DC} = \sigma_{DC}^2 = N_{RO} \cdot \Gamma_{DC}/2\pi \cdot t_D \end{gathered} \quad \text{(S4)}$$

$$\begin{gathered} C_{\Downarrow} \sim \mathcal{N}(\mu, \sigma^2) \\ \mu = N_{RO}(\epsilon + \Gamma_{DC} \cdot t_D) \\ \sigma^2 = N_{RO}(\epsilon \cdot (1-\epsilon) + \Gamma_{DC}/2\pi \cdot t_D) \end{gathered} \quad \text{(S5)}$$

The difference in counts $\delta C = C_{\Downarrow} - C_{\Uparrow}$ is used to measure the state of the nuclear spin. The integral between 0 and ∞ of the distribution for δC is a direct measure of $P_{\Downarrow}$. Since $C_{\Downarrow}$ and $C_{\Uparrow}$ are Gaussian distributions,

$$\begin{aligned} \delta C &\sim \mathcal{N}(\mu - \mu_{DC}, \sigma^2 + \sigma_{DC}^2) \\ P_{\Downarrow} &= \int_0^\infty \frac{e^{-\frac{\left(x-(\mu-\mu_{DC})\right)^2}{2\cdot(\sigma^2+\sigma_{DC}^2)}}}{\sqrt{2\pi\cdot(\sigma^2+\sigma_{DC}^2)}} dx \\ &= \frac{1}{2}\left(1 - \mathrm{Erf}\left[\frac{\mathrm{SNR}}{\sqrt{2}}\right]\right) \end{aligned} \quad \text{(S6)}$$

$$\text{SNR} = \frac{\epsilon}{\sqrt{\epsilon \cdot (1-\epsilon) + 2\Gamma_{DC}/2\pi \cdot t_D}} \cdot \sqrt{N_{RO}}$$

The SNR increases with N_{RO} which leads to an exponential increase of the probability of a correct detection with N_{RO}. The prefactor is calculated from the fluorescence curve of the same measurement, from which the following parameters were obtained, $\epsilon = 0.18$, $\Gamma_{DC} = 150\ \text{s}^{-1}$, $t_D = 2.6$ ms. More details about how these values are obtained are given in section 2.

The cross-relaxation events introduce a dynamic change in the population of the nuclear spin states. Due to the finite probability $\eta^{z,d}$ to relax into the opposite state, the evolution of the population can be modeled via the following rate equations

$$\begin{gathered} \frac{\Delta p_{|\Downarrow\rangle}}{\Delta N_{RO}} = \eta^d p_{|\Uparrow\rangle} - \eta^z p_{|\Downarrow\rangle} \\ \frac{\Delta p_{|\Downarrow\rangle}}{\Delta N_{RO}} = \eta^z p_{|\Downarrow\rangle} - \eta^d p_{|\Uparrow\rangle} \\ p_{|\Downarrow\rangle} + p_{|\Uparrow\rangle} = 1 \end{gathered} \tag{S7}$$

In order to account for preparation errors, the initial condition $p_{|\Downarrow\rangle}(0) = p_0 < 1$ is used. Since $\eta^{z,d} \ll 1$, the dynamics of the system are much slower than the step size of N_{RO} and it can be approximated to $\frac{\Delta p_{|\Downarrow\rangle}}{\Delta N_{RO}} \approx \frac{\Delta p_{|\Downarrow\rangle}}{\Delta N_{RO}}$. Solving the equations

$$p_{|\Downarrow\rangle}(N_{RO}) = \frac{1}{2}\left(p_0 - \frac{\eta^d}{\eta^d+\eta^z}\right) \cdot e^{-(\eta^d+\eta^z)\cdot N_{RO}} + \frac{\eta^d}{\eta^d+\eta^z}, \tag{S8}$$

In the case of Er_5, $\eta^z \approx \eta^d = \eta$ (see Methods 4) and

$$p_{|\Downarrow\rangle}(N_{RO}) = \frac{1}{2}\left(p_0 - \frac{1}{2}\right) \cdot e^{-2\eta\cdot N_{RO}} + \frac{1}{2}, \tag{S9}$$

Since $\text{SNR}(N_{RO}) \gg 2\eta \cdot N_{RO}$ the two processes can be considered independent and the total fidelity of the read-out can be approximated to

$$P_{\Downarrow} = \frac{1}{2}\left(1 - \text{Erf}\left[\frac{\text{SNR}}{\sqrt{2}}\right]\right) p_{|\Downarrow\rangle}(N_{RO}). \tag{S10}$$

An equivalent expression can be found for the complementary process when preparing into $|\Uparrow\rangle$. Fitting the model to the experimental data we obtain p_0 = 0.97 ± 0.02 and η = 3.2 ± 0.2 · 10^{-4} for the cross-relaxation $|\Downarrow\rangle \rightarrow |\Uparrow\rangle$. For the complementary transition, p_0 = 0.94 ± 0.02 and η = 2.6 ± 0.2 · 10^{-4}. Using the relation presented in the main text $\eta = \frac{B^2}{4\omega_I^2}\frac{\kappa^2}{\kappa^2+4\omega_I^2}$ we obtain $B/2\pi$ = 74 ± 7 kHz and $B/2\pi$ = 67 ± 6 kHz respectively. Within error, the two values are similar. However, the measurement does not capture the tail of the exponential reducing the accuracy of the result.

6. Measurement of ω_I

To measure the bare nuclear spin frequency ω_I, we rely on the observed AC-Zeeman shift when driving the forbidden transitions. The undriven frequency difference $\delta_{d,z}^{(0)}$ between ω_I and double and zero-quantum transition frequency can be analytically obtained from the Hamiltonian,

$$\delta_{d,z}^{(0)} = \pm\frac{1}{2}(\omega_I^+ + \omega_I^-),$$
$$\omega_I^+ = \left(\omega_I + \frac{A}{2}\right)\cos\xi_+ - \frac{B}{2}\sin\xi_+,$$
$$\omega_I^- = \left(\omega_I - \frac{A}{2}\right)\cos\xi_- + \frac{B}{2}\sin\xi_-, \qquad \text{(S11)}$$
$$\tan\xi_+ = \frac{-B}{A + 2\omega_I},$$
$$\tan\xi_- = \frac{-B}{A - 2\omega_I}$$

Which in the high-field limit $\omega_S \gg A, B$, this approximates to $\delta_{d,z}^{(0)} = \omega_I$. When resonantly driving the forbidden transitions, the energy levels will be shifted due to the AC-Zeeman effect. The origin of the shift lies in the frequency difference between the drive and the allowed transitions and depends on the amplitude of the drive Ω.

In the case of a simple two-level system, the ac-Zeeman shift is given by $\frac{\Omega^2}{2\Delta}$ where Δ is the detuning of the drive with respect to the transition. In the case of a four-level system, there are two allowed transitions, each detuned by $\Delta_{1,2}$. Each of the two allowed transitions is in turn shifted by $\delta_{1,2} = \frac{\Omega^2}{2\Delta_{1,2}}$. The frequency of the forbidden resonance under drive is therefore

$$\delta_{z,d}(\Omega) = \delta_{z,d}^{(0)} \pm \frac{1}{2}(\delta_1 + \delta_2) = \delta_{z,d}^{(0)} \pm \frac{1}{2}\left(\frac{\Omega^2}{2\Delta_1} + \frac{\Omega^2}{2\Delta_2}\right), \tag{S12}$$

For a resonant drive on the forbidden transition $\Delta_{1,2} = \omega_I^{\pm} - \frac{(\delta_1+\delta_2)}{2}$ (see Fig. S6). Rearranging the terms we obtain that the center of the resonance is

$$\delta_{z,d}(\Omega) = \delta_{z,d}^{(0)} + \frac{\Omega^2}{4}\frac{1}{\omega_I^{+} - \delta_{z,d}(\Omega) + \delta_{z,d}^{(0)}} + \frac{\Omega^2}{4}\frac{1}{\omega_I^{-} - \delta_{z,d}(\Omega) + \delta_{z,d}^{(0)}}. \tag{S13}$$

Evaluating the expression, we obtain

$$\delta_{z,d} = \pm\delta_{z,d}^{(0)} - \frac{\Omega^2}{2}\left(\frac{1}{2\delta_{z,d} + \Delta\omega_I} + \frac{1}{2\delta_{z,d} - \Delta\omega_I}\right). \tag{S14}$$

$$\Delta\omega_I = \omega_I^{+} - \omega_I^{-}.$$

The experimental data presented in Fig. 4E was fitted to this model, using the measured values for Er_5 of $A/2\pi$ = 34.5 kHz and $B/2\pi$ = 103 kHz (see Methods 7), which yields the nuclear spin frequency $\omega_I/2\pi$ = 788.1 $\pm$ 0.4 kHz.

7. Measurement of B via Rabi frequencies

The Rabi frequency for the allowed and forbidden transitions are simply the product of the matrix element of the driving term. Calling B_1 the drive amplitude at resonance, the allowed Rabi frequency is given by

$$\Omega = |\bar{\gamma} \cdot \bar{B}_1| \cdot \langle \downarrow\Uparrow | S\_x | \uparrow\Uparrow \rangle = |\bar{\gamma} \cdot \bar{B}_1| / 2, \tag{S15}$$

since the allowed transitions are resonant with the resonator. On the other hand, the zero- and double-quantum frequencies are detuned by δ. Assuming constant input power, the forbidden Rabi frequency is

$$\Omega_{z,d} = |\bar{\bar{\gamma}} \cdot \bar{B}_1| \cdot \langle \downarrow\Uparrow | S_x | \uparrow\Downarrow \rangle \cdot \frac{1}{\sqrt{1+\frac{4\delta^2}{\kappa^2}}} = \tag{S16}$$

$$= \frac{\Omega}{2} \cdot \left(\frac{B}{2\omega_I - A} + \frac{B}{2\omega_I + A}\right) \cdot \frac{1}{\sqrt{1 + \frac{4\delta^2}{\kappa^2}}}.$$

Therefore, the value of B can be measured by comparing the Rabi frequencies on the allowed and forbidden transitions at a given input power. For the same drive amplitude, Rabi oscillations on the allowed transition are notably faster, since the matrix element is larger. In the measurements presented in Fig. 3, the drive amplitude for the forbidden transition was α^{-1} = 6.2 larger compared to the allowed transition, which needs to be taken into account in the B estimate, as

$$|B| = \alpha \frac{\Omega_{z,d}}{\Omega} \cdot (2\omega_I \pm A) \cdot \sqrt{1 + \frac{4\delta^2}{\kappa^2}} \quad \text{(S17)}$$

The Rabi frequency from the zero- and double-quantum transitions measurements (resp. 104±7 kHz and 102±7 kHz) agree within error. However, there is a notable difference compared to the values obtained through the cross-relaxation method (~ 70 kHz). This is a more precise method compared to the cross-relaxation analysis presented in Methods 4 and 5 as it merely relies on frequency fitting coherent oscillations; therefore, in the calculation performed in Methods 6, we take $B/2\pi$ = 103 ± 7 kHz, the average between the zero- and double-quantum transitions calculation.

8. Dipole-dipole interaction calculation and site assignment

We use the point-dipole approximation to estimate the hyperfine coupling between the Er^{3+} ion and its neighboring nuclear spins. The Hamiltonian for this system is

$$H = \omega_S \cdot S_z' + \omega_I \cdot I_z + H_{dd} \quad \text{(S18)}$$

$$H_{dd} = \frac{\mu_0}{4\pi r^3}\left[\bar{\mu}_S \cdot \bar{\mu}_I - 3r^{-2}(\bar{\mu}_S \cdot \bar{r})(\bar{\mu}_I \cdot \bar{r})\right]$$

where $\bar{\mu}_S = \bar{\bar{\gamma}}_{\mathrm{Er}^{3+}} \cdot \bar{S}$ and $\bar{\mu}_I = \gamma_W \cdot \bar{I}$ are the magnetic moments of the electron and the nuclear spin and $\bar{r}$ is the vector separating the two magnets. Note that the electron spin operators $\bar{S}$ of the dipole-dipole Hamiltonian are not the same compared to the spin operator of the Zeeman term S_z due to the anisotropy of the gyromagnetic tensor $\bar{\bar{\gamma}}_{\mathrm{Er}^{3+}}$. The dipolar perturbation can be expanded in terms of (S_x, S_y, S_z) and (I_x, I_y, I_z). Since the first term of the Hamiltonian is much larger than the rest, the terms containing S_x and S_y can be ignored, which is known as the secular approximation. The resulting Hamiltonian is

$$H = \omega_S \cdot S_z' + \omega_I \cdot I_z + AS_z'I_z + BS_z'I_x. \quad \text{(S19)}$$

The values for A and B depend non-trivially on the orientation ofB_0. These terms were calculated numerically for the 10 tungsten sites in the unit cell centered around the paramagnetic impurity. The magnetic field is applied in the *(y - z)* plane, with the angle between the field and the *z*-axis θ<1º. An angle β = 0.8 ± 0.1º was measured between the c-axis and the *(y - z)* plane, which is taken into consideration when computing the hyperfine dipolar interaction. Figure S7 plots the calculated hyperfine parameters A and B as a function of θ. Table 9 presents the measured hyperfine parameters for the different ions discussed in the main text, as well as the calculated range for the parameters for Type I, II and III. The table is organized as follows, all ions are sorted depending on their Type assignment (see below) and the last column shows the calculated values from a pure dipole-dipole interaction. We only show the minimum and maximum (min – max)

values for A and B in the range of θ that is specified. If the minimum and maximum values are closer than 1 kHz, only the average is given. For $Er_1 - Er_4$, $|B|$ is calculated from the cross-relaxation method (see Methods 4). For Er_5, $|B|$ is obtained from the Rabi sideband method (Methods 7).

For the nuclear spins coupled to Er_2 and Er_4(2), the measured values of A and B are close to those expected from Type III sites, making this site assignment likely. The other nuclear spins have larger values of $|A|$ and $|B|$, implying that they are either Type I or Type II sites; however, the match is not quantitative with any of the sites. Several reasons could explain this. First, local deformations of the crystal could be caused by the larger positive charge of Er^{3+} compared to the original Ca^{2+}. Second, the measured ions are notably strained, as evidenced by the fact that they are found several mT above the center of the ensemble line (*20*). In the case of Er_5, the value of $|B|$ measured is notably larger than the range predicted both for Type I and Type II; however, for larger angles, Type I spins reach such large B values (see Fig. S7), motivating a tentative assignment of Er_5 to a Type I site. We finally note that site assignment should be made easier by the measurement of the angular dependence of A and B.

9. Electron spin spectroscopy and control

The electron spin characteristics for Er_{1-5} are presented in Table 9. The values that are not specified were not measured during the experiment. A representative example is Er_5, presented in Fig. S8. To perform the initial detection of single spins, the fluorescence signal of the sample is measured after applying a 5 μs long square pulse for different values of the magnetic field. When a single spin is in resonance with the superconducting resonator, an increase on the fluorescence signal will be measured after the excitation. This corresponds to every peak in Fig. S8A, where Er_5 is highlighted in red. The lifetime of the spin T_1 is extracted by measuring directly the fluorescence count rate as a function of time after excitation (Fig. S8B). The coupling to the spin resonator g_0 is calculated from the Purcell effect, $\Gamma_R = \frac{4g_0^2}{\kappa}$. We note that g_0 for Er_2 is the highest coupling measured by this technique. Based on the coupling strength we estimate that the ions are within 100 nm of the nanowire (see Methods 1). The coherence times T_2^* and T_2 are measured respectively through a Ramsey and Echo sequence (Fig. S8C & D). The different contrast and dark counts between the Ramsey and Echo measurements are due to different operating points of the SMPD.

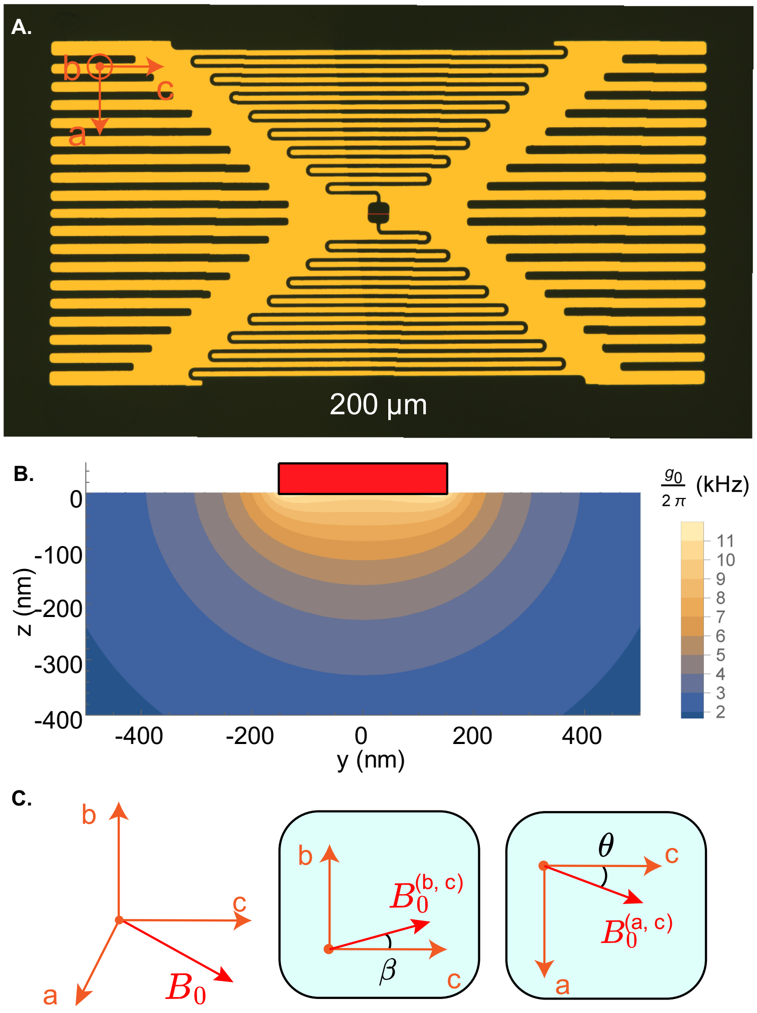

Figure S1: **Micrograph of the sample and magnetic field orientation structure**. **A**. False color micrograph of a Nb thin-film resonator of the same design fabricated on a $CaWO_4$ crystal. The nanowire is colored in red while the rest of the Nb metallization is colored in yellow. The picture is stitched from two independent images. **B**. Spatial map for the coupling g_0 between the Er^{3+} and the superconducting resonator. The map is shown as a transversal cut around the nanowire (in yellow). **C**. Crystalline axis and magnetic field. β and θ are defined as the angle between the c-axis and the projection of B_0 in the (b, c) plane and the (a, c) plane respectively.

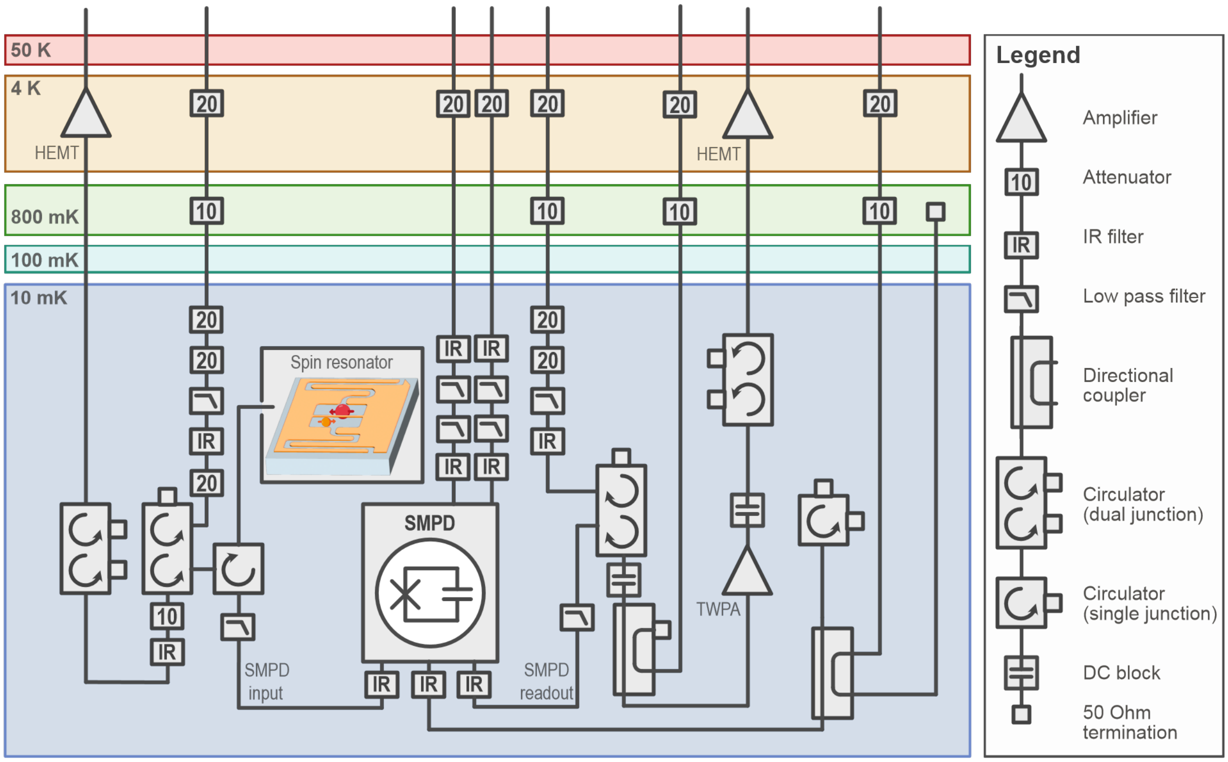

Figure S2: **Schematic of the setup**. Wiring and components of the experimental setup within the dilution fridge. The SMPD is hosted inside of a magnetic shield to protect it from the stray magnetic field from the vector magnet and external magnetic fluctuations.

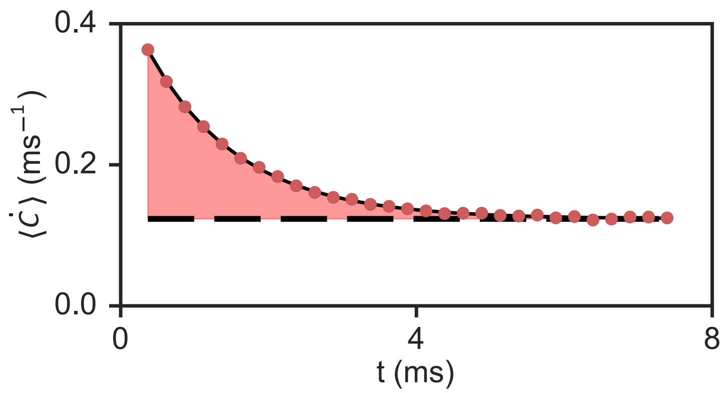

Figure S3: **Spin-efficiency and dark-counts measurement for** Er_5. Measured (dots) fluorescence count rate is fitted with an exponential decay (solid black line). The offset of the decay corresponds to the SMPD dark-count rate Γ_{DC}= 0.12 counts/ms (dashed black line). The spin-efficiency η = 0.4 is the integral (red area) of the exponential decay.

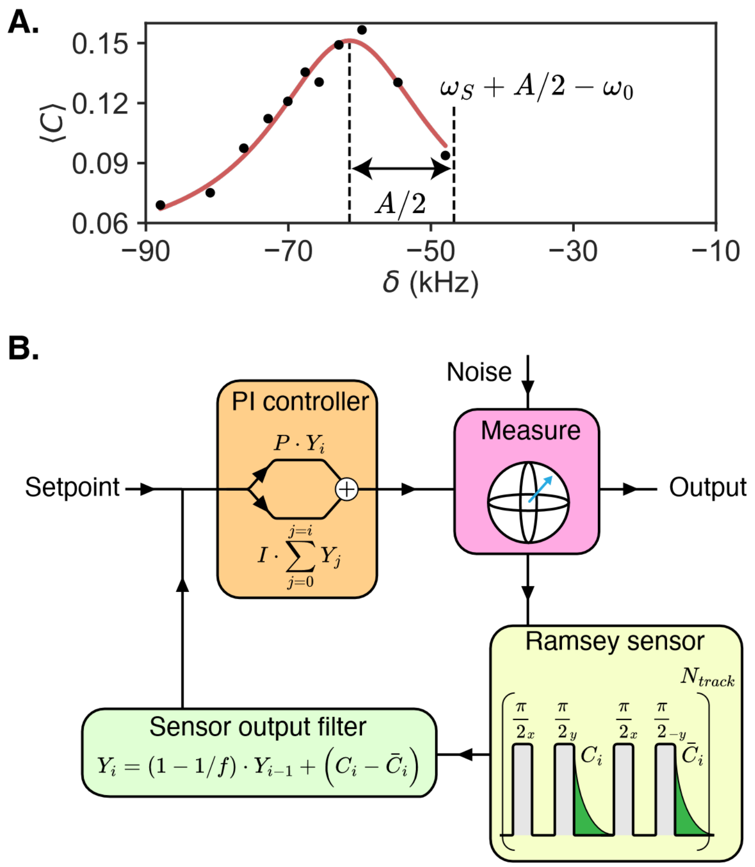

Figure S4: **Spectral tracking measurement and feedback loop diagram**. **A**. Spectral tracking sample measurement. Measured (dots) ensemble averaged number of counts as a function of δ after pumping the zero-quantum transition of Er5. A Lorentzian fit (solid line) yields the center of the EPR resonance (left dashed vertical line). ω_S is calculated by subtracting $A/2$ (right dashed vertical line). **B**. Feedback loop diagram. The PI loop is run after every measurement to correct any shift in the frequencies of the system. After a given iteration of the measurement i, the Ramsey sensor measures $C_i - \bar{C}_i$, a proxy quantity for the detuning of the system. The sensor is filtered to obtain Y_i. Finally, the detuning of the system is corrected via a PI controller before the next measurement starts.

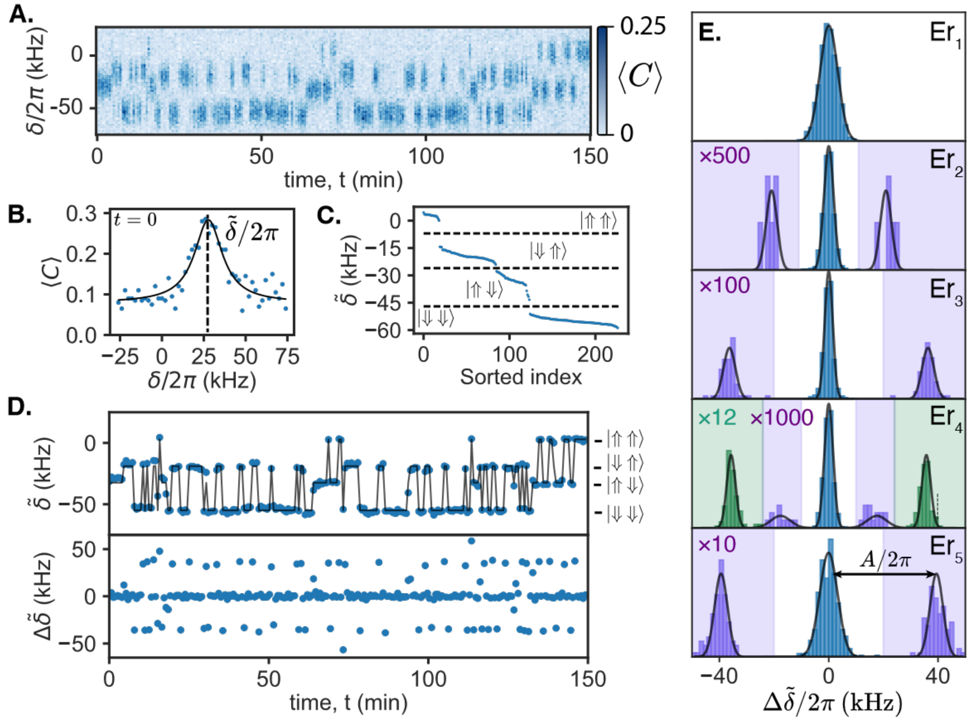

Figure S5: **Trace analysis steps**. **A.** High resolution spectra as a function of time for Er_4. **B.** Spectrum at time equal to 0 seconds (blue dots) and Lorentzian fit (black line) with center $\tilde{\delta}$ (dashed black line). **C.** Sorted values of $\tilde{\delta}$ (blue dots) and derivative bound thresholds (dashed black lines). Each sector corresponds to the nuclear spins state that is indicated. **D.** *top*. Lorentzian fit center $\tilde{\delta}$ (blue dots) as a function of time and nuclear spin state assignment (black line). *bottom*. Difference between consecutive values of $\tilde{\delta}$ as a function of time. **E.** Histograms of $\Delta\tilde{\delta}$ for the five ions. The colored areas have been enhanced by a factor as indicated. Solid lines are Gaussian fits to each peak. The difference between their centers yields the longitudinal hyperfine coupling constant of each nuclear spin, $A/2\pi$.

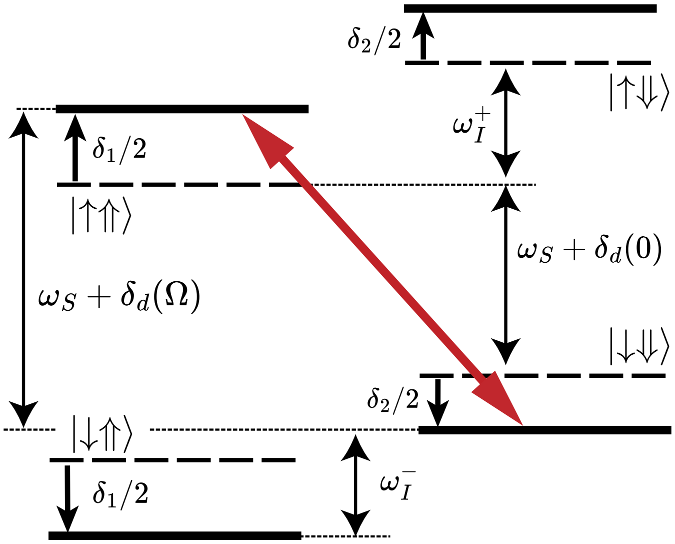

Figure S6: **AC-Zeeman shift of the energy level diagram under off-resonant drive.** The spin levels are frequency-shifted by the microwave drive, from their $\Omega = 0$ value (black dashed lines) to the value under drive (solid black line). Microwave drive of amplitude Ω_d is shown as a red double arrow.

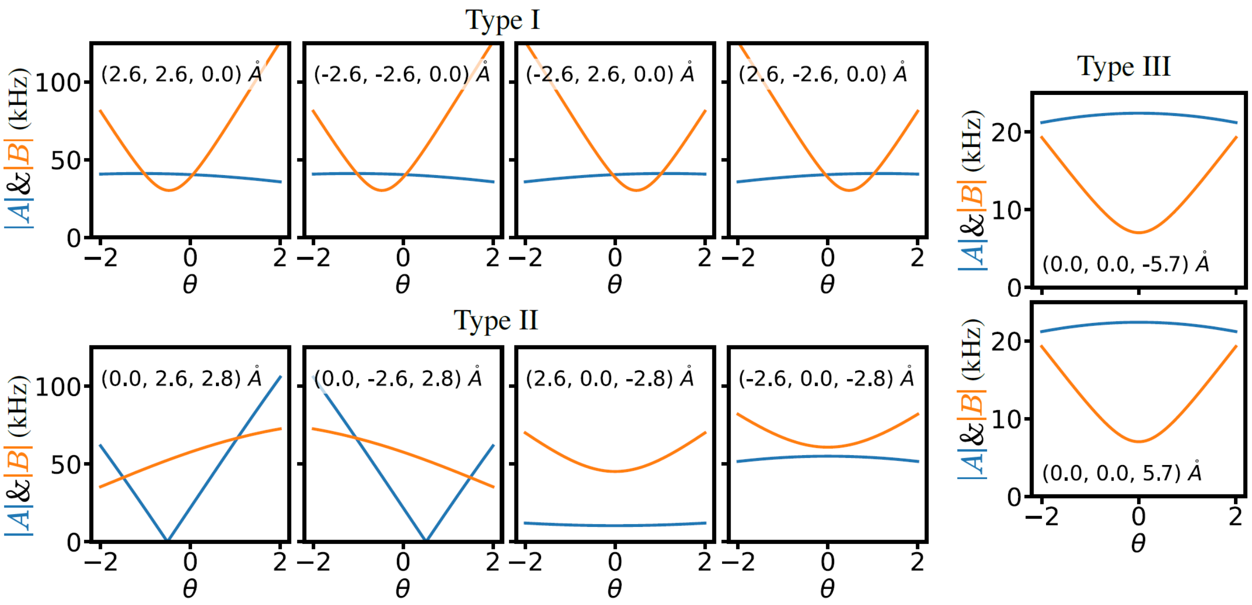

Figure S7: **Hyperfine parameters for Types I, II and III nuclear spins as a function of θ.** Hyperfine coupling A and B obtained through a dipole-dipole Hamiltonian simulation. The values are shown as a function of the angle between the projection of B_0 in the (a, c)-plane and the c-axis of the crystal, θ. The calculation considers the measured misalignment β = 0.8. The position of the ions is specified in angstroms. The plots are organized depending on the relative position of the nuclear spin with respect to the Er^{3+} ion.

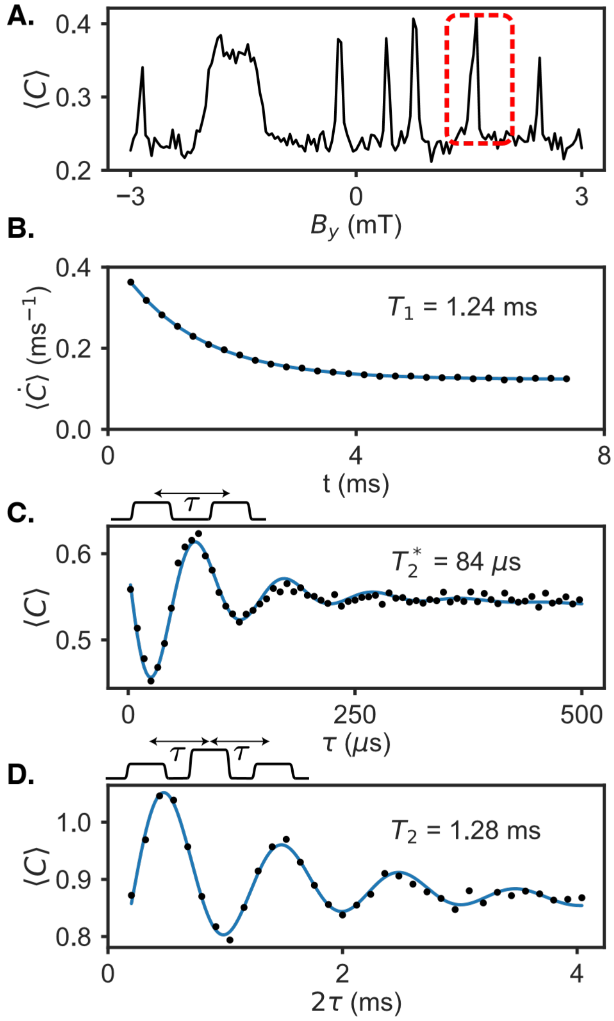

Figure S8: **Electron spin characterization**. **A.** Ensemble averaged counts as a function of magnetic field B_y. For this measurement the Z and X magnets are set to persistent mode and B_z = 446.2 mT and B_x = 0.4 mT. Each peak corresponds to a single Er^{3+} ion. Er_5 is highlighted in red. **B.** Electron spin fluorescence. Count rate after exciting the spin with a π-pulse. The signal decays with T_1 = 1.24 ms to a flat background from the dark counts of the detector. **C.** Ramsey measurement. The coherence of the electron spin is $T_2^* = 0.84$. The measurement introduces an arbitrary 1 kHz detuning to the recovery pulse of the sequence for ease of fitting **D.** Echo measurement. The coherence of the electron spin is increased to $T_2 = 1.28$ ms after one dynamical decoupling step.

	Er_2	Er_3	$Er_4^{(1)}$	$Er_4^{(2)}$	Er_5
$\eta^d \cdot 10^5$	1.5±0.7	5.8±0.6	18±0.2	1.6±0.7	24±2
$\eta^z \cdot 10^5$	0.8±0.3	4.2±0.4	9±0.1	0.7±3	24±2

Table S1: Cross-relaxation probability measured through the time-trace analysis. The uncertainties of the cross-relaxation probabilities are estimated through the Wald method with a 1 sigma confidence interval.

Type I	Er_3	$Er_4^{(2)}$	Er_5	calc.
θ (º)	-0.2	-0.1	0.1	-0.2 – 0.1
$\|A\|/2\pi$ (kHz)	36.3±0.6	35.8±0.5	39.6±0.5	40
$\|B\|/2\pi$ (kHz)	27±3	40±5	103±3	35 – 48

Type II	Er_3	$Er_4^{(2)}$	calc.
θ (º)	-0.2	-0.1	-0.3 – -0.1
$\|A\|/2\pi$ (kHz)	36.3±0.6	35.8±0.5	12 – 57
$\|B\|/2\pi$ (kHz)	27±3	40±5	45 – 61

Type III	Er_2	$Er_4^{(1)}$	calc.
θ (º)	-0.3	-0.1	-0.3 – -0.1
$\|A\|/2\pi$ (kHz)	21±1	19±1	23
$\|B\|/2\pi$ (kHz)	12±3	11±3	8

Table S2: Hyperfine parameters for the different Erbium ions and the angle θ at which the measurements were taken. Ions are organized as per their Type assignment. Three values are given for B of Er_5, obtained from the spectroscopic trace analysis, read-out probability fit, and Rabi driving respectively. The last column gives the ranges of the hyperfine coupling values from the dipole-dipole calculation (see text).

	Er_1	Er_2	Er_3	Er_4	Er_5
T_1 (ms)	2.0±0.3	0.71±0.1	0.90±0.1	0.80±0.3	1.24±0.1
$g_0/2\pi$ (kHz)	3.6±0.2	6.0±0.2	5.3±0.2	5.6±0.2	4.5±0.1
T_2^* (μs)	-	53±5	-	170±5	81±2
T_2 (ms)	-	-	-	2.05±0.03	1.3±0.1

Table S3: Electron spin parameters for the different ions presented on the main text. Dashes are in place when the measurement was not performed.
